# Traxoprodil Produces Antidepressant-Like Behaviors in Chronic Unpredictable Mild Stress Mice through BDNF/ERK/CREB and AKT/FOXO/Bim Signaling Pathway

**DOI:** 10.1155/2023/1131422

**Published:** 2023-02-10

**Authors:** Yahui Wang, Zehuai Liang, Wenyue Song, Yu Qin, Noah Todd, Mingqi Gao

**Affiliations:** ^1^School of Pharmacy, China Medical University, Shenyang 110122, China; ^2^School of Medicine, Indiana University, Fort Wayne 46805, USA

## Abstract

Traxoprodil is a selective N-methyl-d-aspartate receptor subunit 2B (NR2B) receptor inhibitor with rapid and long-lasting antidepressant effects. However, the appropriate dosage, duration of administration, and underlying mechanism of traxoprodil's antidepressant effects remain unclear. The purpose of this study is to compare the antidepressant effects of traxoprodil in different doses and different durations of administration and to explore whether traxoprodil exerts antidepressant effects via the brain-derived neurotrophic factor/extracellular signal-regulated kinase/cAMP-response element binding protein (BDNF/ERK/CREB) and protein kinase B/Forkhead box O/building information modelling (AKT/FOXO/Bim) signaling pathway. Mice were randomly divided into control group, chronic unpredictable mild stress (CUMS) + vehicle group, CUMS + traxoprodil (10 mg/kg, 20 mg/kg, and 40 mg/kg) groups, and CUMS + fluoxetine (5 mg/kg) group, followed by a forced swimming test, tail suspension test, and sucrose preference test. Western blotting and immunohistochemistry were used to measure the protein expression of BDNF, p-ERK1/2, p-CREB, NR2B, AKT, FOXO1, FOXO3a, and Bim. Compared with the control group, CUMS treatment increased immobility time; decreased sucrose preference; reduced expression of BDNF, p-ERK1/2, and p-CREB; and increased expression of AKT, FOXO, and Bim in the hippocampus. These alterations were ameliorated by administration of 20 mg/kg or 40 mg/kg of traxoprodil after 7 or 14 days of administration and with 10 mg/kg of traxoprodil or 5 mg/kg of fluoxetine after 21 days of administration. At the 7-day and 14-day timepoints, traxoprodil displayed dose-dependent antidepressant effects, with 20 and 40 mg/kg doses of traxoprodil producing rapid and strong antidepressant effects. However, at 21 days of administration, 10 and 20 mg/kg doses of traxoprodil exerted more pronounced antidepressant effects. The mechanism of traxoprodil's antidepressant effects may be closely related to the BDNF/ERK/CREB and AKT/FOXO/Bim signaling pathway.

## 1. Introduction

Depression is a common mood disorder characterized by low mood, anhedonia, anxiety, and sleep disorders, and it is accompanied by changes in neurotransmitters in the brain [1]. It is ranked by WHO as the single largest contributor to global disability and the major contributor to suicide deaths [2]. Depression seriously affects human health and quality of life and causes a heavy burden to families and society [3]. The unclear pathogenesis of depression and the delayed onset of action and low remission rate of traditional antidepressants urge us to explore new antidepressants [4]. A growing body of research indicates that glutamate neurotransmitters are involved in the occurrence and development of depression, and the glutamate system has become one of the hotspots for novel antidepressant development [5, 6].

Glutamate, as the major excitatory neurotransmitter in the central nervous system (CNS), plays an important role in learning, memory, and synaptic plasticity in physiological states [7]. Multiple pieces of evidence suggest that the N-methyl-D-aspartate (NMDA) receptor (NMDAR), an ionotropic glutamate receptor, is closely associated with the pathogenesis of depression and that NMDA receptor antagonists such as ketamine and MK-801 can induce rapid and sustained antidepressant effects [8–11]. NR2B is a subunit of the NMDA receptor. Studies have noted the selective NR2B receptor antagonists such as Ro 25-6981, MK-0657, and traxoprodil produce rapid and significant antidepressant effects with fewer undesirable adverse effects [12–14].

The neurotrophic hypothesis holds that brain-derived neurotrophic factor (BDNF) regulates the function of neurons by activating tyrosine kinase receptor B (TrkB) and mitogen-activated protein kinase (MAPK) signaling pathways [15, 16]. The extracellular signal-regulated kinase 1/2 (ERK1/2) is a crucial protein in MAPK signal cascades involved in the development of depression [17]. As a transcription factor, cAMP response element-binding protein (CREB) plays an important role in neurogenesis and neuronal plasticity associated with the pathogenesis of depression and could be activated by MAPK kinase signal pathway (MEK/ERK) [18]. Research indicates that the expression of CREB and BDNF in depression models is reduced compared with control groups, and these changes can be reversed by administration of the NMDA receptor antagonist memantine [19]. In addition, studies reported that ketamine, Ro 25-6981, and other NMDA receptor antagonists also reverse the deficit in phosphorylation of ERK1/2 and CREB in hippocampus of rodents exposed to social defeat stress or chronic unpredictable stress [12, 20–22]. Meanwhile, the forkhead box O (FOXO) transcription factors mediate cell death in a variety of diseases and regulate the expression of BH3-only member of Bcl-2 family (Bim), which induces neuronal apoptosis. Although FOXO is mostly involved in cell cycle regulation in the pancreas [23], it has also been shown to regulate neurons in the hippocampus. Thus, we hypothesized that FOXO transcription factors may also be involved in the development of depression.

**Figure 1:**
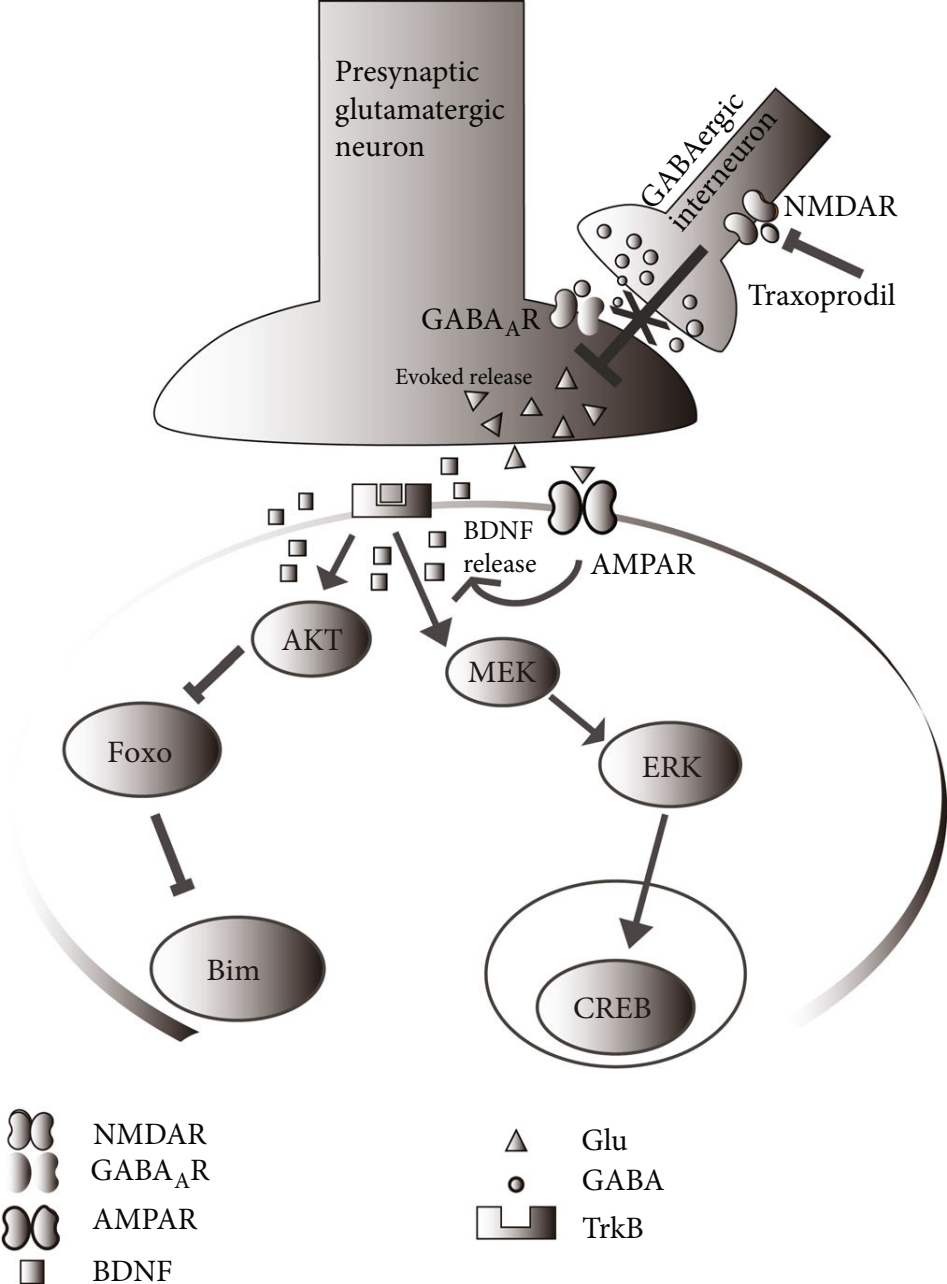
Molecular pathway of BDNF/ERK/CREB and AKT/FOXO/Bim.

Traxoprodil is a selective NR2B receptor inhibitor. Administration of a subactive dose of traxoprodil with subactive doses of desipramine, paroxetine, and other traditional antidepressants at subtherapeutic doses led to an antidepressant-like effect in mice [24]. A randomized, placebo-controlled, double-blind study showed that a single dose of traxoprodil significantly improved depressive symptoms and maintained response status for at least 1 week after infusion [25]. The main goal of the current study was to assess the antidepressant effects of traxoprodil at different concentrations and at different durations of therapy in chronic mild and unpredictable mild stress model mice and determine whether traxoprodil exerts its antidepressant effects through activation of the BDNF/ERK/CREB and AKT/FOXO/Bim signaling pathways (Figure 1).

## 2. Methods and Materials

### 2.1. Animals

Male CD1 mice (5-6 weeks) were obtained from Liaoning Changsheng Biotechnology Co., Ltd. (Liaoning, China) and kept under standard laboratory conditions (temperature 25 ± 2°C and humidity 45-55%) with a 12 h light/dark cycle (light on: 7 : 00-19 : 00). Mice were provided with food and water freely except during behavioral tests or during a water and food deprivation stressor. All the experimental procedures were done in accordance with the Guide for The Care and Use of Laboratory Animals, National Institutes of Health, and were approved by the Experimental Animal Welfare and Ethics Committee of China Medical University (IACUC Issue No. CMU2019167). All efforts were taken to minimize animal suffering and reduce the number of animals used.

### 2.2. Chronic Unpredictable Mild Stress (CUMS) Procedure

Mice were randomly divided into a control group (*n* = 6), CUMS + vehicle group (*n* = 6), CUMS + traxoprodil (10 mg/kg (*n* = 6), 20 mg/kg (*n* = 6), and 40 mg/kg (*n* = 6)) groups, and a CUMS + fluoxetine (5 mg/kg) group (*n* = 6). The CUMS procedure was based on the methods as described previously. In brief, all mice except the control group were exposed to different stressors: (1) illumination overnight, (2) cage tilted 45° for 12 h, (3) wet bedding for 12 h, (4) no padding for 12 h, (5) restraint for 4 h, and (6) water and food deprivation for 24 h. The CUMS procedure lasted for 3 weeks, and the stressors were applied daily in a random order to add the unpredictable characteristic of the experiment [26–29].

### 2.3. Drug Administration

The traxoprodil (Sigma-Aldrich, St. Louis, USA) doses were dissolved in 0.9% saline containing 1% Tween-80, and the fluoxetine hydrochloride (Sigma-Aldrich, St. Louis, USA) doses were dissolved in 0.9% saline. Mice were administered traxoprodil (10 mg/kg, 20 mg/kg, and 40 mg/kg, i.p.), fluoxetine hydrochloride (5 mg/kg, i.p.), or 0.9% saline for 7, 14, and 21 days, respectively. All drug solutions used in this study were administered at a volume of 10 ml/kg and prepared just before use.

### 2.4. Forced Swimming Test (FST)

After treatment, the FST was performed on mice in a separate, quiet environment. The mice were placed individually in a glass cylinder (height 25 cm and diameter 16 cm) containing 20 cm of water (23-25°C) and adapted for 10 min the day before the test. All animals were forced to swim for 6 min, and their immobility time was recorded during the last 4 min of the test. A mouse was judged to be immobile when it stopped struggling and floated in the water or made only necessary movements to keep its head above the water.

### 2.5. Tail Suspension Test (TST)

The TST was performed the day after the FST. In short, the mice were attached individually with adhesive tape 1.5 cm from the tip of the tail and suspended 15 cm above a table for 6 min. After 2 min of acclimatization, the immobility time of each mouse in the last 4 min was observed and recorded. Immobility time was defined as the duration in which a mouse did not struggle, bend, or twist.

### 2.6. Sucrose Preference Test (SPT)

The SPT lasted for 4 days. The first 3 days were devoted to training the mice, and testing occurred on the 4th day. On the first day, mice were housed individually and given two bottles of 1% sucrose solution (*w*/*v*, 100 ml) for 24 h. On the second day, one bottle of 1% sucrose solution was replaced with pure water for 24 h, and the position of the sucrose solution bottle and pure water bottle was changed at 12 h to avoid drinking position preference. On the third day, the mice were deprived of food and water for 24 h. Next, mice were given free access to a preweighed bottle of 1% sucrose solution (100 ml) and a preweighed bottle of pure water (100 ml). After 12 hours, the consumption of sucrose solution and pure water was recorded, and sucrose preference (%) was calculated by the following formula: Here SP(%) = sucrose solution consumption (g)/(sucrose solution consumption (g) + pure water consumption (g)) × 100%.

### 2.7. Western Blotting

After the mice were decapitated, the hippocampus was dissected and immediately homogenized in RIPA buffer (Beyotime, Shanghai, China) containing PMSF (Beyotime, Shanghai, China, PMSF : RIPA = 1 : 100) for 30 min on ice and then centrifugated at 14000 rpm for 25 min at 4°C. Total protein supernatant was collected, and the concentration was determined via BCA protein assay kit (Beyotime, Shanghai, China). Equal amounts of protein were loaded per well on 12% SDS-PAGE gels and then transferred onto PVDF membranes. After blocking in 5% skim milk or BSA for 1-2 hours at room temperature, the membranes were incubated at 4°C overnight with anti-BDNF antibody (1 : 1000; Abcam, Cambridge, UK), anti-p-ERK1/2 antibody (1 : 1000; Cell Signaling Technology, MA, USA), anti-ERK1/2 antibody (1 : 1000; Cell Signaling Technology, MA, USA), anti-p-CREB antibody (1 : 1000; Cell Signaling Technology, MA, USA), anti-CREB antibody (1 : 1000; Cell Signaling Technology, MA, USA), anti-NR2B antibody (1 : 1000; Abcam, Cambridge, UK), anti-AKT antibody (1 : 1000; Cell Signaling Technology, MA, USA), anti-FOXO1 antibody (1 : 1000; Cell Signaling Technology, MA, USA), anti-FOXO3a antibody (1 : 1000; Cell Signaling Technology, MA, USA), anti-Bim (1 : 1000; Cell Signaling Technology, MA, USA), or anti-*β*-actin antibody (1 : 5000; Proteintech, Chicago, USA). The PVDF membranes were washed and incubated at room temperature with goat anti-rabbit or goat anti-mouse IgG HRP-conjugated antibody (1 : 5000; ABclonal, Wuhan, China) for 2 hours. The protein bands were visualized by enhanced ECL kit (Beyotime, Shanghai, China) in a gel image analyzing system. The quantification of protein bands was performed using ImageJ software and normalized against the level of *β*-actin protein.

### 2.8. Immunohistochemistry (IHC)

The brains of mice were fixed with 4% paraformaldehyde and embedded in paraffin and then cut into sections with thickness of 4 *μ*m using a paraffin slicer. Slices were boiled in a 10 mM citrate buffer (pH 6.0) for 10 min to expose antigens and then cooled to room temperature. The endogenous peroxides of sections were quenched with 0.3% H_2_O_2_ for 10 min. Slices were blocked with 5% normal goat serum for 10 min and then incubated with anti-BDNF antibody (1 : 200; Abcam, Cambridge, UK), anti-p-ERK1/2 antibody (1 : 200; Cell Signaling Technology, MA, USA), anti-p-CREB antibody (1 : 200; Cell Signaling Technology, MA, USA), anti-AKT antibody (1 : 200; Cell Signaling Technology, MA, USA), or anti-FOXO3a antibody (1 : 200; Cell Signaling Technology, MA, USA) overnight at 4°C. The resulting slides were washed and incubated with biotinylated secondary antibody (1 : 300; Proteintech, Chicago, USA) for 2 h at room temperature. Then, sections were stained with 3,3-diaminobenzidine (DAB, Zhongshan Jinqiao, Beijing, China) for 1-2 min, counterstained with hematoxylin for 20 s, decolorized, and sealed. Finally, the hippocampal subfields (CA1 and hilus) were observed under a 400x light microscope (Nikon Instruments Inc., Tokyo, Japan) and analyzed using ImageJ software.

### 2.9. Statistical Analysis

All data were expressed as mean ± SEM, analyzed, and plotted using GraphPad Prism 8.0 (GraphPad Software, Inc., San Diego, CA, USA). Significant differences were compared using one-way analysis of variance (ANOVA). *P* < 0.05 was considered statistically significant.

## 3. Results

### 3.1. Effects of Traxoprodil Treatment on the FST and TST in CUMS Mice

**Figure 2:**
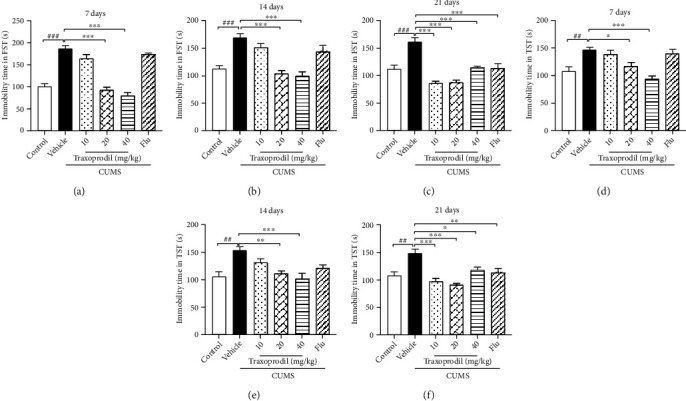
Effects of 10, 20, and 40 mg/kg traxoprodil administration on immobility time in the FST (7 d (a); 14 d (b); 21 d (c)) and TST (7 d (d); 14 d (e); 21 d (f)) after 7, 14, and 21 days, respectively. Data are expressed as mean ± SEM (*n* = 6). ^##^*P* < 0.01 and ^###^*P* < 0.001 vs. control group; ^∗^*P* < 0.05, ^∗∗^*P* < 0.01, and ^∗∗∗^*P* < 0.001 vs. CUMS-vehicle group.

After applications of the chronic unpredictable mild stressor protocol, the immobility times of the FST (7 d, 14 d, and 21 d: *P* < 0.001, respectively) and the TST (7 d: *P* < 0.01; 14 d: *P* < 0.01; and 21 d: *P* < 0.01) in the CUMS model groups were significantly increased at 7, 14, and 21 days of treatment compared to controls. Treatment with 20 or 40 mg/kg of traxoprodil significantly decreased the immobility times in the FST (Figure 2(a); 20 mg/kg: *P* < 0.001 and 40 mg/kg: *P* < 0.001) and TST (Figure 2(d); 20 mg/kg: *P* < 0.05 and 40 mg/kg: *P* < 0.001) in CUMS mice after 7 days of administration, but the 10 mg/kg traxoprodil group and 5 mg/kg fluoxetine group did not demonstrate such changes. After 14 days of administration, traxoprodil given at doses of 20 or 40 mg/kg significantly reduced the immobility times of the FST (Figure 2(b); 20 mg/kg: *P* < 0.001 and 40 mg/kg: *P* < 0.001) and TST (Figure 2(e); 20 mg/kg: *P* < 0.01 and 40 mg/kg: *P* < 0.001). The immobility time of CUMS mice in the 10 mg/kg traxoprodil group and fluoxetine group was slightly decreased at the 14-day timepoint, but the difference was not statistically significant. Furthermore, our results indicated that the immobility time during the FST (Figure 2(c); 10, 20, and 40 mg/kg of traxoprodil: *P* < 0.001, respectively) and TST (Figure 2(f); 10 mg/kg traxoprodil: *P* < 0.001; 20 mg/kg traxoprodil: *P* < 0.001; 40 mg/kg traxoprodil: *P* < 0.05; and fluoxetine: *P* < 0.01) was reduced by the administration of traxoprodil at 10, 20, and 40 mg/kg or fluoxetine after 21-day treatment, but the immobility time in the 40 mg/kg traxoprodil group and 5 mg/kg fluoxetine group was longer than that in the 10 and 20 mg/kg traxoprodil groups at the same timepoint.

### 3.2. Effects of Traxoprodil on the Sucrose Preference in the CUMS Mice

**Figure 3:**
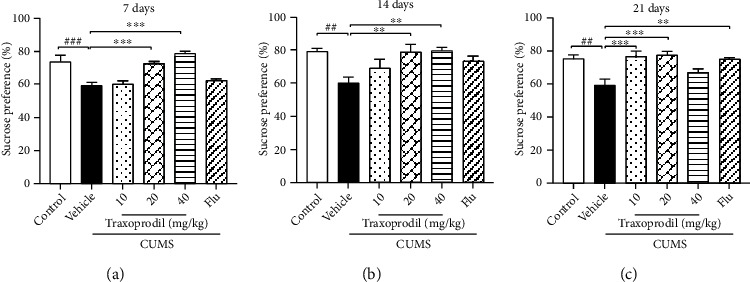
Effects of traxoprodil administration for 7 (a), 14 (b), or 21 (c) days on sucrose preference in the SPT. Data are represented as mean ± SEM (*n* = 6). ^##^*P* < 0.01 and ^###^*P* < 0.001 vs. control group; ^∗∗^*P* < 0.01 and ^∗∗∗^*P* < 0.001 vs. CUMS-vehicle group.

As shown in Figure 3, CUMS treatment induced a statistically significant reduction in the sucrose preference in all three dosing groups (7 d: *P* < 0.001; 14 d: *P* < 0.01; and 21 d: *P* < 0.01). After 7 days of administration, 20 (*P* < 0.001) and 40 (*P* < 0.001) mg/kg of traxoprodil significantly increased sucrose consumption in CUMS mice, but 5 mg/kg of fluoxetine and 10 mg/kg of traxoprodil did not (Figure 3(a)). This increase in sucrose preference was also sustained after 14 days of treatment in the 20 (*P* < 0.01) and 40 (*P* < 0.01) mg/kg traxoprodil groups (Figure 3(b)). Although the sucrose consumption of 5 mg/kg fluoxetine and 10 mg/kg traxoprodil groups was slightly increased after 14 days, the difference did not reach significance. Finally, 10 (*P* < 0.001) and 20 (*P* < 0.001) mg/kg traxoprodil treatment led to significantly increased sucrose preference after 21 days, as did 5 mg/kg fluoxetine (*P* < 0.01), but 40 mg/kg of traxoprodil did not (Figure 3(c)).

### 3.3. Effects of Traxoprodil Administration on Total Protein Levels of BDNF, p-ERK1/2, and p-CREB in the Hippocampus

**Figure 4:**
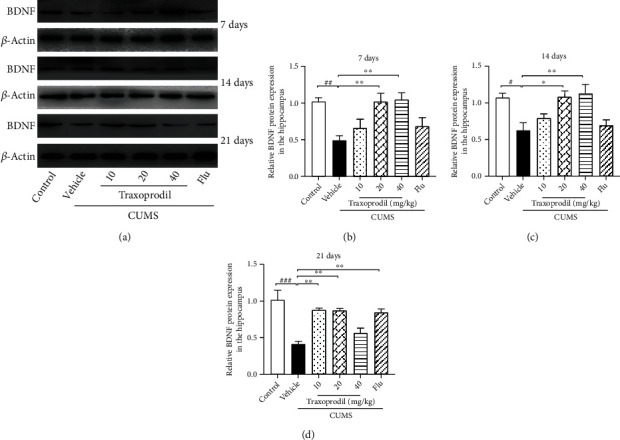
Effects of traxoprodil administration for 7 (b), 14 (c), or 21 (d) days on the expression of BDNF in the hippocampus (a). Data are represented as mean ± SEM (*n* = 6). ^##^*P* < 0.01 and ^###^*P* < 0.001 vs. control group; ^∗^*P* < 0.05, ^∗∗^*P* < 0.01, and ^∗∗∗^*P* < 0.001 vs. CUMS-vehicle group.

**Figure 5:**
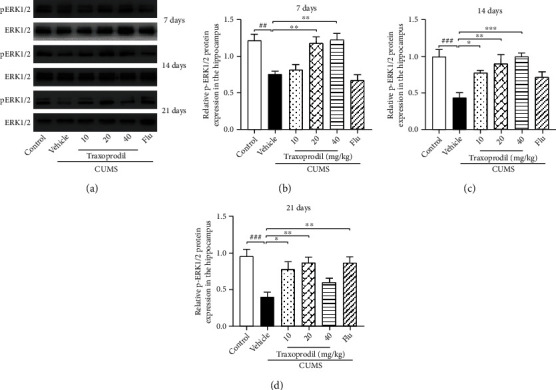
Effects of traxoprodil administration for 7 (b), 14 (c), or 21 (d) days on the expression of p-ERK1/2 in the hippocampus (a). Data are represented as mean ± SEM (*n* = 6). ^##^*P* < 0.01 and ^###^*P* < 0.001 vs. control group; ^∗^*P* < 0.05, ^∗∗^*P* < 0.01, and ^∗∗∗^*P* < 0.001 vs. CUMS-vehicle group.

**Figure 6:**
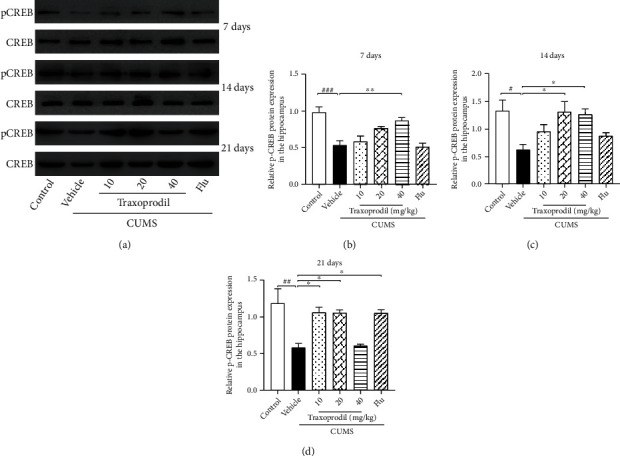
Effects of traxoprodil administration for 7 (b), 14 (c), or 21 (d) days on the expression of p-CREB in the hippocampus (a). Data are represented as mean ± SEM (*n* = 6). ^##^*P* < 0.01 and ^###^*P* < 0.001 vs. control group; ^∗^*P* < 0.05, ^∗∗^*P* < 0.01, and ^∗∗∗^*P* < 0.001 vs. CUMS-vehicle group.

After 7 days of administration, western blot analysis showed that the protein expression levels of BDNF (Figure 4(b), *P* < 0.01, *F*(5, 30) = 6.032), p-ERK1/2 (Figure 5(b), *P* < 0.01, *F*(5, 30) = 11.27), and p-CREB (Figure 6(b), *P* < 0.001, *F*(5, 30) = 10.98) in the CUMS model group were significantly decreased compared with the control group, using *β*-actin, ERK1/2, and CREB as inner controls, respectively. The expression of BDNF (Figure 4(b); 20 mg/kg: *P* < 0.01 and 40 mg/kg: *P* < 0.01) and p-ERK1/2 (Figure 5(b); 20 mg/kg: *P* < 0.01 and 40 mg/kg: *P* < 0.01) in the hippocampus was significantly increased in the 20 and 40 mg/kg traxoprodil groups. The expression level of p-CREB in the hippocampus of CUMS mice was significantly increased in the 40 mg/kg traxoprodil group (Figure 6(b); *P* < 0.01), but the increase in the 20 mg/kg traxoprodil group did not reach significance. Neither 10 mg/kg traxoprodil nor 5 mg/kg fluoxetine influenced the expression of BDNF, p-ERK1/2, or p-CREB in the hippocampus of CUMS mice after 7 days of treatment.

At 14 days of administration, CUMS treatment significantly reduced the expression of BDNF (Figure 4(c), *P* < 0.05, *F*(5, 18) = 6.567), p-ERK1/2 (Figure 5(c), *P* < 0.001, *F*(5, 30) = 7.192), and p-CREB (Figure 6(c), *P* < 0.05, *F*(5, 30) = 4.303) in the hippocampus compared with the control group. Traxoprodil at 20 and 40 mg/kg significantly increased the hippocampal protein levels of BDNF (Figure 4(c); 20 mg/kg: *P* < 0.05 and 40 mg/kg: *P* < 0.01), p-ERK1/2 (Figure 5(c); 20 mg/kg: *P* < 0.01 and 40 mg/kg: *P* < 0.001), and p-CREB (Figure 6(c); 20 mg/kg: *P* < 0.05 and 40 mg/kg: *P* < 0.05), while 10 mg/kg of traxoprodil only induced a significant increase in p-ERK1/2 (*P* < 0.05) expression. BDNF and p-CREB levels in the hippocampus were slightly increased in the 10 mg/kg traxoprodil group and 5 mg/kg fluoxetine group, but the differences did not reach significance.

After 21 days of administration, CUMS mice displayed significant reductions in hippocampal BDNF (Figure 4(d), *P* < 0.001, *F*(5, 12) = 11.07), p-ERK1/2 (Figure 5(d), *P* < 0.001, *F*(5, 30) = 6.128), and p-CREB (Figure 6(d), *P* < 0.01, *F*(5, 12) = 7.746) compared to the control group. Nevertheless, 21-day treatment with fluoxetine and 10 and 20 mg/kg doses of traxoprodil markedly upregulated the expression of BDNF (Figure 4(d); 10 mg/kg traxoprodil: *P* < 0.01; 20 mg/kg traxoprodil: *P* < 0.01; and fluoxetine: *P* < 0.01), p-ERK1/2 (Figure 5(d); 10 mg/kg traxoprodil: *P* < 0.05; 20 mg/kg traxoprodil: *P* < 0.01; and fluoxetine: *P* < 0.01), and p-CREB (Figure 6(d); 10 mg/kg traxoprodil: *P* < 0.05; 20 mg/kg traxoprodil: *P* < 0.05; and fluoxetine: *P* < 0.05), but treatment with 40 mg/kg of traxoprodil did not.

### 3.4. Effects of Traxoprodil Administration on Total Protein Levels of NR2B, AKT, FOXO1, FOXO3a, and Bim in the Hippocampus

**Figure 7:**
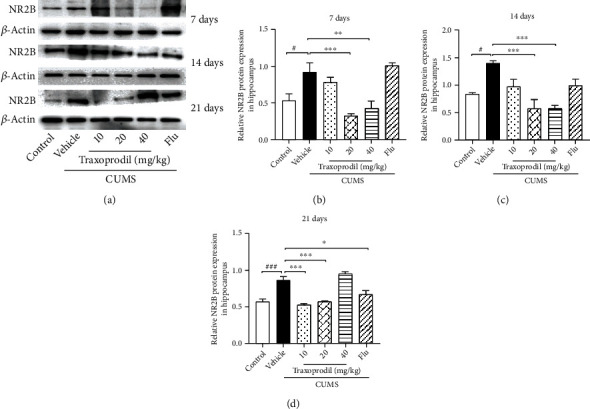
Effects of traxoprodil administration for 7 (b), 14 (c), or 21 (d) days on the expression of NR2B in the hippocampus (a). Data are represented as mean ± SEM (*n* = 3). ^###^*P* < 0.001 vs. control group; ^∗^*P* < 0.05, ^∗∗^*P* < 0.01, and ^∗∗∗^*P* < 0.001 vs. CUMS-vehicle group.

**Figure 8:**
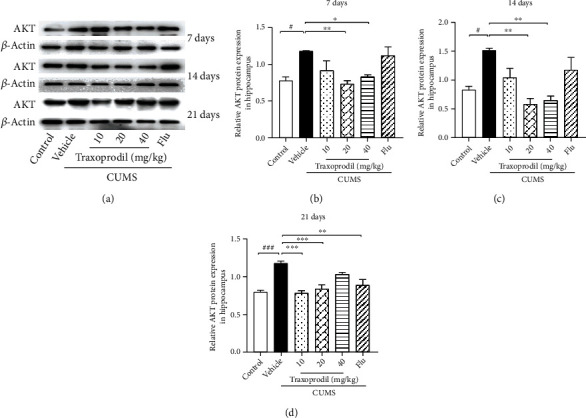
Effects of traxoprodil administration for 7 (b), 14 (c), or 21 (d) days on the expression of AKT in the hippocampus (a). Data are represented as mean ± SEM (*n* = 3). ^###^*P* < 0.001 vs. control group; ^∗∗∗^*P* < 0.001 vs. CUMS-vehicle group.

**Figure 9:**
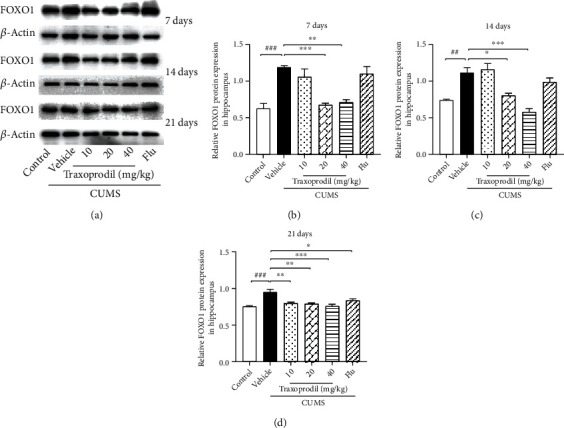
Effects of traxoprodil administration for 7 (b), 14 (c), or 21 (d) days on the expression of FOXO1 in the hippocampus (a). Data are represented as mean ± SEM (*n* = 3). ^###^*P* < 0.001 vs. control group; ^∗∗^*P* < 0.01 and ^∗∗∗^*P* < 0.001 vs. CUMS-vehicle group.

**Figure 10:**
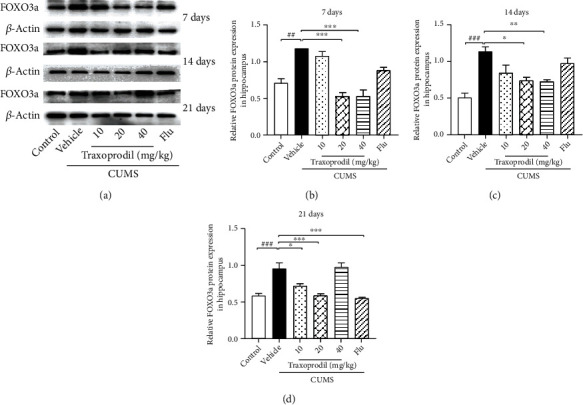
Effects of traxoprodil administration for 7 (b), 14 (c), or 21 (d) days on the expression of FOXO3a in the hippocampus (a). Data are represented as mean ± SEM (*n* = 3). ^##^*P* < 0.01 and ^###^*P* < 0.001 vs. control group; ^∗∗^*P* < 0.01 and ^∗∗∗^*P* < 0.001 vs. CUMS-vehicle group.

**Figure 11:**
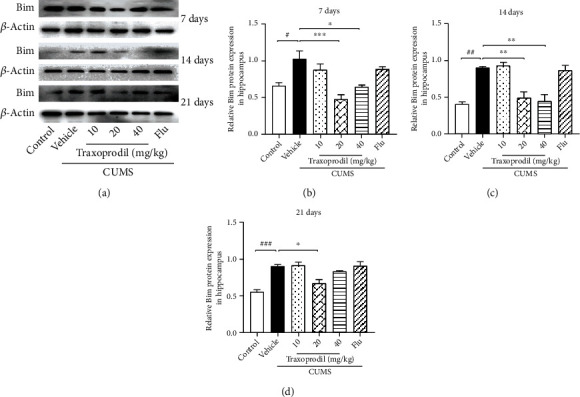
Effects of traxoprodil administration for 7 (b), 14 (c), or 21 (d) days on the expression of Bim in the hippocampus (a). Data are represented as mean ± SEM (*n* = 3). ^##^*P* < 0.01 and ^###^*P* < 0.001 vs. control group; ^∗∗^*P* < 0.01 and ^∗∗∗^*P* < 0.001 vs. CUMS-vehicle group.

To determine whether the AKT/FOXO pathway is involved in the effects of CUMS on depression-like behaviors, western blotting was used to examine the expression of related proteins in the hippocampus. After 7 days of administration, our results revealed a significant increase in the protein levels of NR2B (Figure 7(b), *P* < 0.05, *F*(5, 18) = 11.60), AKT (Figure 8(b), *P* < 0.05, *F*(5, 18) = 5.811), FOXO1 (Figure 9(b), *P* < 0.001, *F*(5, 18) = 13.22), FOXO3a (Figure 10(b), *P* < 0.01, *F*(5, 12) = 19.83), and Bim (Figure 11(b), *P* < 0.05, *F*(5, 12) = 9.878) in the CUMS group compared with the control group. The 20 and 40 mg/kg traxoprodil groups reduced the protein levels of NR2B (Figure 7(b); 20 mg/kg: *P* < 0.001 and 40 mg/kg: *P* < 0.01), AKT (Figure 8(b); 20 mg/kg: *P* < 0.01 and 40 mg/kg: *P* < 0.05), FOXO1 (Figure 9(b); 20 mg/kg: *P* < 0.001 and 40 mg/kg: *P* < 0.01), FOXO3a (Figure 10(b); 20 mg/kg: *P* < 0.001 and 40 mg/kg: *P* < 0.001), and Bim (Figure 11(b); 20 mg/kg: *P* < 0.001 and 40 mg/kg: *P* < 0.05) in CUMS mice. However, there was no significant difference in the 10 mg/kg traxoprodil and 5 mg/kg fluoxetine groups compared with the CUMS group.

After 14 days of administration, CUMS increased NR2B (Figure 7(c), *P* < 0.05, *F*(5, 18) = 9.373), AKT (Figure 8(c), *P* < 0.05, *F*(5, 18) = 8.178), FOXO1 (Figure 9(c), *P* < 0.01, *F*(5, 18) = 17.36), FOXO3a (Figure 10(c), *P* < 0.001, *F*(5, 12) = 11.25), and Bim (Figure 11(c), *P* < 0.01, *F*(5, 12) = 15.82) protein levels, whereas traxoprodil at 20 and 40 mg/kg significantly reduced the protein levels of NR2B (Figure 7(c); 20 mg/kg: *P* < 0.001 and 40 mg/kg: *P* < 0.001), AKT (Figure 8(c); 20 mg/kg: *P* < 0.01 and 40 mg/kg: *P* < 0.01), FOXO1 (Figure 9(c); 20 mg/kg: *P* < 0.05 and 40 mg/kg: *P* < 0.001), FOXO3a (Figure 10(c); 20 mg/kg: *P* < 0.05 and 40 mg/kg: *P* < 0.01), and Bim (Figure 11(c); 20 mg/kg: *P* < 0.01 and 40 mg/kg: *P* < 0.01) in CUMS mice. There was no significant difference in levels of NR2B, AKT, FOXO1, FOXO3a, or Bim in the 10 mg/kg traxoprodil and 5 mg/kg fluoxetine groups.

After 21 days of administration, CUMS mice displayed significant increases in hippocampal NR2B (Figure 7(d), *P* < 0.001, *F*(5, 12) = 29.30), AKT (Figure 8(d), *P* < 0.001, *F*(5, 12) = 14.26), FOXO1 (Figure 9(d), *P* < 0.001, *F*(5, 12) = 11.14), FOXO3a (Figure 10(d), *P* < 0.001, *F*(5, 12) = 18.44), and Bim (Figure 11(d), *P* < 0.001, *F*(5, 12) = 13.88) compared to the control group. However, the 5 mg/kg fluoxetine group and 10 and 20 mg/kg traxoprodil groups decreased the expression of NR2B (Figure 7(d); fluoxetine: *P* < 0.05; 10 mg/kg traxoprodil: *P* < 0.001; and 20 mg/kg traxoprodil: *P* < 0.001), AKT (Figure 8(d); fluoxetine: *P* < 0.01; 10 mg/kg traxoprodil: *P* < 0.001; and 20 mg/kg traxoprodil: *P* < 0.001), FOXO1 (Figure 9(d); fluoxetine: *P* < 0.05; 10 mg/kg traxoprodil: *P* < 0.01; and 20 mg/kg traxoprodil: *P* < 0.01), and FOXO3a (Figure 10(d); fluoxetine: *P* < 0.001; 10 mg/kg traxoprodil: *P* < 0.05; and 20 mg/kg traxoprodil: *P* < 0.001). Although 40 mg/kg traxoprodil significantly reduced FOXO1 (Figure 9(d); *P* < 0.001) protein level, it did not significantly alter levels of any other protein compared with the CUMS group. Levels of Bim were only significantly decreased in the 20 mg/kg traxoprodil group (Figure 11(d); *P* < 0.05).

### 3.5. Effects of Traxoprodil Administration on the Expression of BDNF, p-ERK1/2, and p-CREB in the Hippocampal CA1 and DG Regions

**Figure 12:**
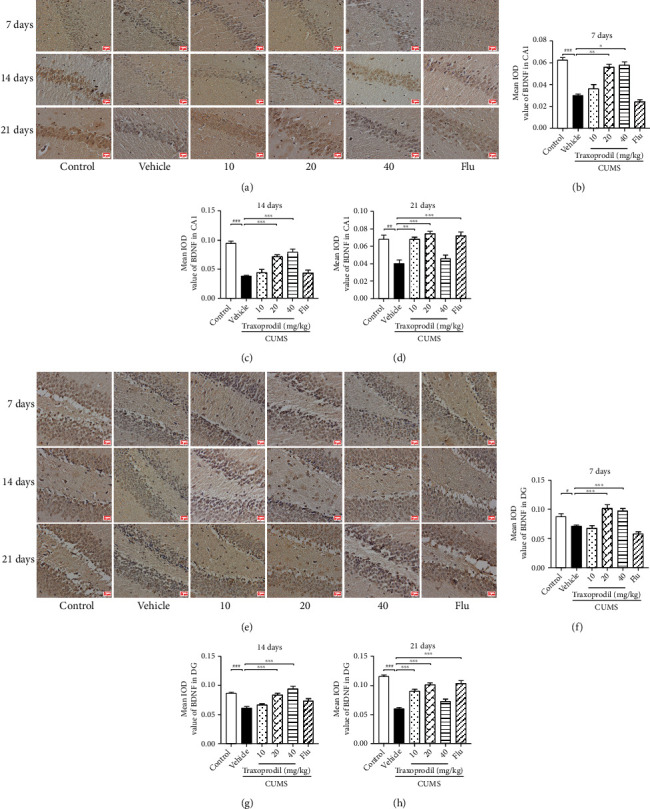
Effects of traxoprodil administration for 7, 14, or 21 days on the expression of BDNF in hippocampal CA1 (a) and DG (e) regions. Average optical density values of BDNF in the CA1 (7 d (b); 14 d (c); 21 d (d)) and DG (7 d (f); 14 d (g); 21 d (h)) regions of the hippocampus are shown after 7, 14, and 21 days of administration. Data are represented as mean ± SEM (*n* = 4). ^#^*P* < 0.05, ^##^*P* < 0.01, and ^###^*P* < 0.001 vs. control group; ^∗^*P* < 0.05, ^∗∗^*P* < 0.01, and ^∗∗∗^*P* < 0.001 vs. CUMS-vehicle group.

**Figure 13:**
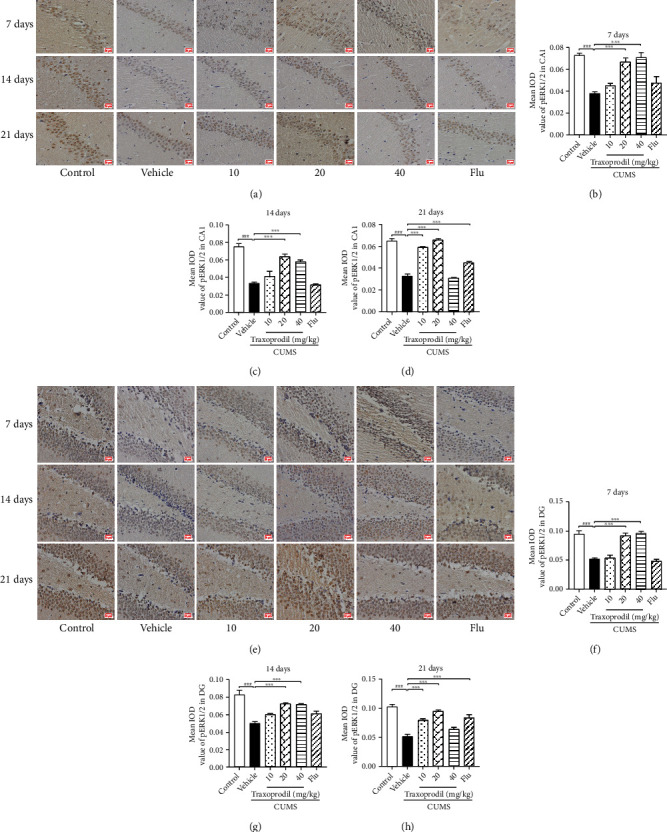
Effects of traxoprodil administration for 7, 14, or 21 days on the expression of p-ERK1/2 in hippocampal CA1 (a) and DG (e) regions. Average optical density values of p-ERK1/2 in the CA1 (7 d (b); 14 d (c); 21 d (d)) and DG (7 d (f); 14 d (g); 21 d (h)) regions of the hippocampus are shown after 7, 14, and 21 days of administration. Data are represented as mean ± SEM (*n* = 4). ^###^*P* < 0.001 vs. control group; ^∗∗∗^*P* < 0.001 vs. CUMS-vehicle group.

**Figure 14:**
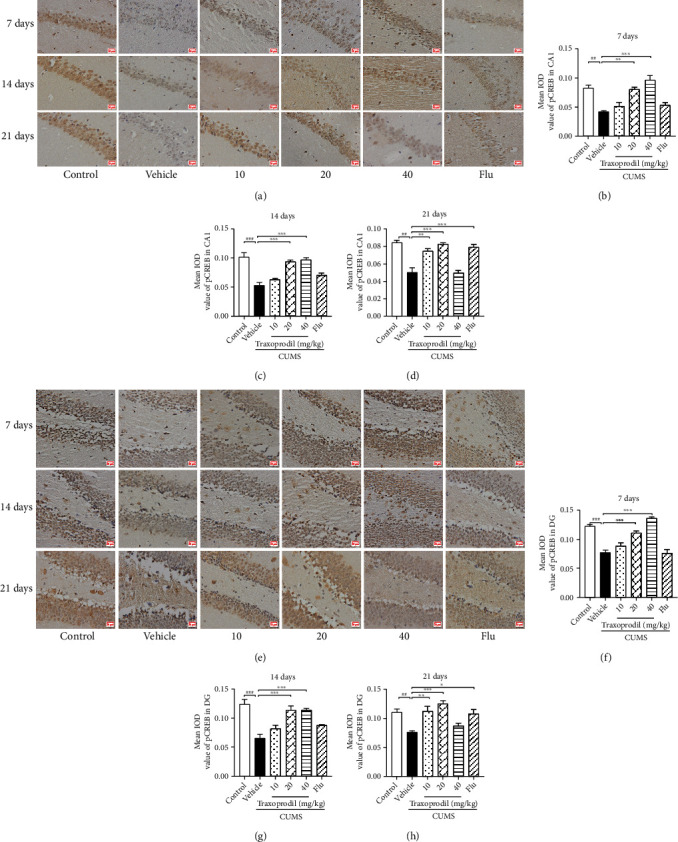
Effects of traxoprodil administration for 7, 14, or 21 days on the expression of p-CREB in hippocampal CA1 (a) and DG (e) regions. The average optical density values of p-CREB in the CA1 (7 d (b); 14 d (c); 21 d (d)) and DG (7 d (f); 14 d (g); 21 d (h)) regions of the hippocampus after 7, 14, and 21 days of administration. Data are represented as mean ± SEM (*n* = 4). ^##^*P* < 0.01 and ^###^*P* < 0.001 vs. control group; ^∗^*P* < 0.05, ^∗∗^*P* < 0.01, and ^∗∗∗^*P* < 0.001 vs. CUMS-vehicle group.

Immunohistochemistry showed that in the hippocampal CA1 and DG regions, there was lower expression of BDNF (CA1: *P* < 0.001, *F*(5, 12) = 40.74 and DG: *P* < 0.05, *F*(5, 18) = 15.68), p-ERK1/2 (CA1: *P* < 0.001, *F*(5, 12) = 18.53and DG: *P* < 0.001, *F*(5, 18) = 35.84), and p-CREB (CA1: *P* < 0.01, *F*(5, 12) = 17.62 and DG: *P* < 0.001, *F*(5, 18) = 37.37) in the CUMS group compared to the control group. However, these changes in expression of BDNF (Figure 12(b); CA1 of 20 mg/kg: *P* < 0.01 and CA1 of 40 mg/kg: *P* < 0.05; Figure 12(f); DG of 20 mg/kg: *P* < 0.001 and DG of 40 mg/kg: *P* < 0.001), p-ERK1/2 (Figure 13(b); CA1 of 20 mg/kg: *P* < 0.001 and CA1 of 40 mg/kg: *P* < 0.001; Figure 13(f); DG of 20 mg/kg: *P* < 0.001 and DG of 40 mg/kg: *P* < 0.001), and p-CREB (Figure 14(b); CA1 of 20 mg/kg: *P* < 0.01 and CA1 of 40 mg/kg: *P* < 0.001; Figure 14(f); DG of 20 mg/kg: *P* < 0.001 and DG of 40 mg/kg: *P* < 0.001) were reversed with the administration of traxoprodil at 20 and 40 mg/kg doses for 7 days. No significant differences were found in the 5 mg/kg fluoxetine and 10 mg/kg traxoprodil groups.

After 14 days of administration, compared with the control group, the protein levels of BDNF (CA1: *P* < 0.001, *F*(5, 12) = 35.61 and DG: *P* < 0.001, *F*(5, 18) = 19.19), p-ERK1/2 (CA1: *P* < 0.001, *F*(5, 12) = 34.90 and DG: *P* < 0.001, *F*(5, 18) = 19.05), and p-CREB (CA1: *P* < 0.001, *F*(5, 12) = 21.84 and DG: *P* < 0.001, *F*(5, 18) = 15.06) in the hippocampal CA1 and DG regions were significantly reduced in the CUMS mice. Treatment with 20 and 40 mg/kg of traxoprodil significantly increased the levels of BDNF (Figure 12(c); CA1 of 20 mg/kg: *P* < 0.001 and CA1 of 40 mg/kg: *P* < 0.001; Figure 12(g); DG of 20 mg/kg: *P* < 0.001 and DG of 40 mg/kg: *P* < 0.001), p-ERK1/2 (Figure 13(c); CA1 of 20 mg/kg: *P* < 0.001 and CA1 of 40 mg/kg: *P* < 0.001; Figure 13(g); DG of 20 mg/kg: *P* < 0.001 and DG of 40 mg/kg: *P* < 0.001), and p-CREB (Figure 14(c); CA1 of 20 mg/kg: *P* < 0.001 and CA1 of 40 mg/kg: *P* < 0.001; Figure 14(g); DG of 20 mg/kg: *P* < 0.001 and DG of 40 mg/kg: *P* < 0.001) in the CA1 and DG regions of the hippocampus in CUMS mice. The protein levels in the 10 mg/kg traxoprodil group and 5 mg/kg fluoxetine group were slightly changed, but no significant differences were found.

After 21 days of administration, CUMS treatment resulted in decreased expression of BDNF (CA1: *P* < 0.01, *F*(5, 12) = 16.28 and DG: *P* < 0.001, *F*(5, 18) = 45.68), p-ERK1/2 (CA1: *P* < 0.001, *F*(5, 12) = 16.5 and DG: *P* < 0.001, *F*(5, 18) = 30.14), and p-CREB (CA1: *P* < 0.01, *F*(5, 12) = 25.14 and DG: *P* < 0.01, *F*(5, 18) = 9.952) in the hippocampus compared with the control group. However, traxoprodil at 10 and 20 mg/kg doses as well as 5 mg/kg fluoxetine significantly elevated the BDNF (Figure 12(d); CA1 of 10 mg/kg traxoprodil: *P* < 0.01; CA1 of 20 mg/kg traxoprodil: *P* < 0.001; and CA1 of fluoxetine: *P* < 0.001; Figure 12(h); DG of 10 mg/kg traxoprodil: *P* < 0.001; DG of 20 mg/kg traxoprodil: *P* < 0.001; and DG of fluoxetine: *P* < 0.001), p-ERK1/2 (Figure 13(d); CA1 of 10 mg/kg traxoprodil: *P* < 0.001; CA1 of 20 mg/kg traxoprodil: *P* < 0.001; and CA1 of fluoxetine: *P* < 0.001; Figure 13(h); DG of 10 mg/kg traxoprodil: *P* < 0.001; DG of 20 mg/kg traxoprodil: *P* < 0.001; and DG of fluoxetine: *P* < 0.001), and p-CREB (Figure 14(d); CA1 of 10 mg/kg traxoprodil: *P* < 0.01; CA1 of 20 mg/kg traxoprodil: *P* < 0.001; and CA1 of fluoxetine: *P* < 0.001; Figure 14(h); DG of 10 mg/kg traxoprodil: *P* < 0.01; DG of 20 mg/kg traxoprodil: *P* < 0.001; and DG of fluoxetine: *P* < 0.05) protein levels in the hippocampus. However, in the 40 mg/kg traxoprodil group, no significant differences were observed compared to CUMS + vehicle mice.

### 3.6. Effects of Traxoprodil Administration on the Expression of FOXO3a and AKT in the Hippocampus

**Figure 15:**
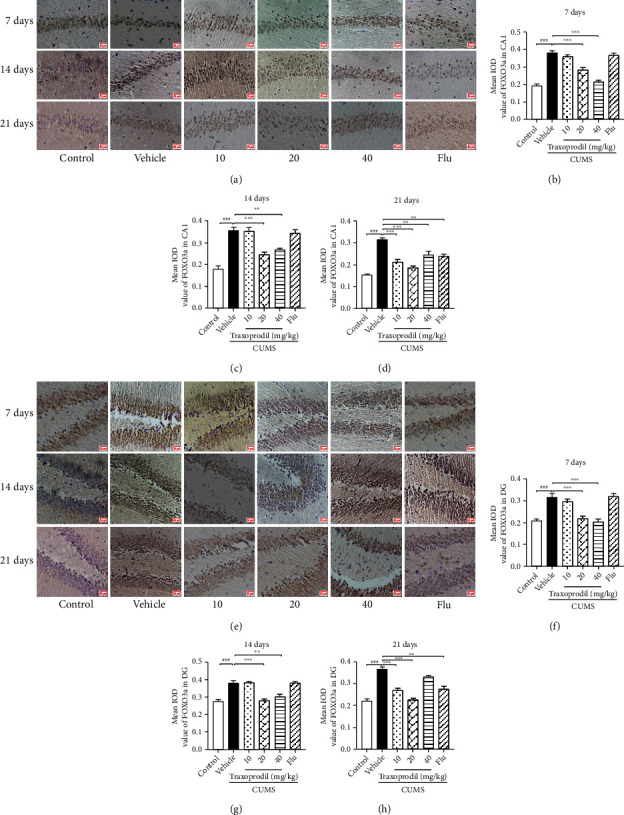
Effects of traxoprodil administration for 7, 14, or 21 days on the expression of FOXO3a in hippocampal CA1 (a) and DG (e) regions. Average optical density values of FOXO3a in the CA1 (7 d (b); 14 d (c); 21 d (d)) and DG (7 d (f); 14 d (g); 21 d (h)) regions of the hippocampus are shown after 7, 14, and 21 days of administration. Data are represented as mean ± SEM (*n* = 3). ^###^*P* < 0.001 vs. control group; ^∗∗^*P* < 0.01 and ^∗∗∗^*P* < 0.001 vs. CUMS-vehicle group.

Molecules of FOXO are important factors to regulation of several cellular functions. Excessive production of FOXO can lead to neuronal cell death through an apoptosis pathway. Current research has shown that the transcriptional activity of FOXO3a is mediated by the PI3K/AKT pathway and other signal pathways. As shown in Figure 15, an increase of FOXO3a (CA1: *P* < 0.001 and DG: *P* < 0.001) expression in the CUMS administration compared to the control group was determined in hippocampal CA1 and DG regions. Traxoprodil at 20 and 40 mg/kg doses reversed this change after 7 (Figure 15(b); CA1 of 20 mg/kg: *P* < 0.001 and CA1 of 40 mg/kg: *P* < 0.001; Figure 15(f); DG of 20 mg/kg: *P* < 0.001 and DG of 40 mg/kg: *P* < 0.001) and 14 days of treatment (Figure 15(c); CA1 of 20 mg/kg: *P* < 0.001 and CA1 of 40 mg/kg: *P* < 0.01; Figure 15(g); DG of 20 mg/kg: *P* < 0.001 and DG of 40 mg/kg: *P* < 0.01), but no significant differences were found in the 5 mg/kg fluoxetine and 10 mg/kg traxoprodil groups. After 21 days of administration, traxoprodil and fluoxetine treatments significantly decreased the levels of FOXO3a (Figure 15(d); CA1 of fluoxetine: *P* < 0.01; CA1 of 10 mg/kg traxoprodil: *P* < 0.001; CA1 of 20 mg/kg traxoprodil: *P* < 0.001; and CA1 of 40 mg/kg traxoprodil: *P* < 0.01; Figure 15(h); DG of 10 mg/kg traxoprodil: *P* < 0.001; DG of 20 mg/kg traxoprodil: *P* < 0.001; and DG of fluoxetine: *P* < 0.01) in CUMS mouse hippocampus, except that treatment with 40 mg/kg of traxoprodil had no effect on FOXO3a level in the DG region of the hippocampus.

**Figure 16:**
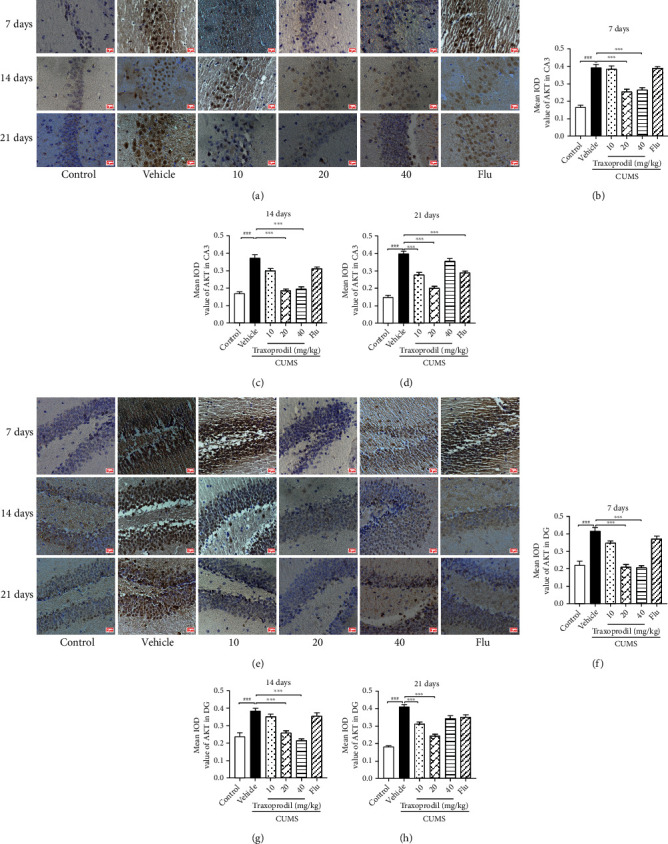
Effects of traxoprodil administration for 7, 14, or 21 days on the expression of AKT in hippocampal CA3 (a) and DG (e) regions. Average optical density values of AKT in the CA3 (7 d (b); 14 d (c); 21 d (d)) and DG (7 d (f); 14 d (g); 21 d (h)) regions of the hippocampus are shown after 7, 14, and 21 days of administration. Data are represented as mean ± SEM (*n* = 3). ^###^*P* < 0.001 vs. control group; ^∗∗^*P* < 0.01 and ^∗∗∗^*P* < 0.001 vs. CUMS-vehicle group.

As presented in Figure 16, compared with the control group, the expression of AKT (CA3: *P* < 0.001 and DG: *P* < 0.001) in the CA3 and DG regions of the hippocampus was significantly increased in the CUMS group. After 7 days of administration, 20 and 40 mg/kg of traxoprodil decreased the levels of AKT in the hippocampus (Figure 16(b); CA3 of 20 mg/kg: *P* < 0.001 and CA3 of 40 mg/kg: *P* < 0.001; Figure 16(f); DG of 20 mg/kg: *P* < 0.001 and DG of 40 mg/kg: *P* < 0.001) and 14 (Figure 16(c); CA3 of 20 mg/kg: *P* < 0.001 and CA3 of 40 mg/kg: *P* < 0.001; Figure 16(g); DG of 20 mg/kg: *P* < 0.001 and DG of 40 mg/kg: *P* < 0.001), but no difference was found in the 5 mg/kg fluoxetine and 10 mg/kg traxoprodil groups. After 21 days of administration, traxoprodil at 10 and 20 mg/kg doses as well as 5 mg/kg fluoxetine significantly decreased the AKT (Figure 16(d); CA3 of fluoxetine: *P* < 0.001; CA3 of 10 mg/kg: *P* < 0.001; and CA3 of 20 mg/kg: *P* < 0.001; Figure 16(h); DG of 10 mg/kg: *P* < 0.001 and DG of 20 mg/kg: *P* < 0.001) protein levels in the hippocampus. However, in the 40 mg/kg traxoprodil group, no significant differences were observed compared to CUMS + vehicle mice.

## 4. Discussion

Current antidepressants are primarily based on altering monoaminergic signaling mechanisms, but delayed onset and treatment resistance limit their clinical use in many patients [30]. Traxoprodil is a selective NR2B receptor antagonist that has been shown to produce a rapid and robust antidepressant response after a single dose without a dissociative reaction [25]. However, additional studies are warranted to explore the antidepressant effects of traxoprodil in different concentrations and durations of treatment. The FST and TST, also known as behavioral despair models, have become the most widely used models for assessing the effects of antidepressants due to strong reliability, high predictive efficacy, and easy operation [31, 32]. Consistent with previous literature [33, 34], our study showed that immobility time during the FST and TST was prolonged in CUMS mice compared to control mice. Meanwhile, the SPT has been widely used to assess anhedonia-like behavior in the CUMS model to indicate the degree of anhedonia in animals [34, 35]. Anhedonia is defined as the loss of ability to feel pleasure and constitutes a prominent feature of depression that is used to assess depressive behavior in preclinical studies. In accordance with previous reports [36, 37], the results of this study showed that compared with the control group, the sucrose preference in the CUMS mice was significantly reduced.

In this study, the traditional antidepressant fluoxetine was used as a positive control drug to evaluate the antidepressant effects of traxoprodil at different concentrations and days of administration. We found that traxoprodil at 10 mg/kg for 21 days significantly reduced immobility time during the FST and TST in mice, similar to the antidepressant effect of fluoxetine at 5 mg/kg. Administration of traxoprodil at 20 and 40 mg/kg for 7 or 14 days significantly improved depressive-like behaviors, indicating that traxoprodil produced rapid and robust antidepressant effects in the CUMS mice. However, we did not observe any significant difference in depressive behaviors of the 10 mg/kg traxoprodil or 5 mg/kg fluoxetine groups compared with the CUMS group at 7 and 14 days. Thus, these results indicate that doses of 20 and 40 mg/kg of traxoprodil elicit rapid and significant antidepressant effects in CUMS mice. Similar to fluoxetine, the administration of traxoprodil at 10 mg/kg showed antidepressant effects only after more than 2 weeks. Additionally, the adverse psychiatric reactions caused by excessive drug accumulation may lead to a decrease in the effect of 40 mg/kg of traxoprodil after 21 days of administration and thereby negatively affect its antidepressant efficacy.

One of the main points of this study was to investigate whether traxoprodil exerts antidepressant effects through the BDNF/ERK/CREB signaling pathway. A large number of previous studies have shown that chronic stress can lead to depression and may be associated with impairment of structural plasticity and neural cellular resilience [37–39]. BDNF is one of the most widely distributed and extensively studied neurotrophic factors in the mammalian brain. BDNF has been implicated in the development of depression, and its expression is influenced by the activity of many antidepressants [40]. Studies have shown that CUMS and other stimuli reduce the expression of BDNF mRNA and protein in the hippocampus and prefrontal cortex, while long-term use of antidepressants significantly increases the expression of BDNF [41]. In this study, the expression of BDNF in the hippocampus of the CUMS group was significantly decreased, but this decrease was reversed after treatment with 10 mg/kg of traxoprodil or 5 mg/kg of fluoxetine for 21 days and with 20 mg/kg or 40 mg/kg of traxoprodil for 7 days or 14 days. These results suggest that the increased expression of BDNF in the hippocampus may be related to the antidepressant effects of traxoprodil. Multiple reports have found that ERK1/2 and CREB modulate neuronal function by activating intracellular signaling cascades [42, 43]. Studies have also indicated that NMDA receptor antagonists such as ketamine and memantine can upregulate the level of BDNF and increase the activation of ERK1/2 and CREB in the hippocampus of animal models [44–46]. Therefore, the phosphorylated protein expression of ERK1/2 and CREB in the hippocampus of each group was measured in this study. Our present data show that the expression levels of phosphorylated ERK1/2 (P-ERK1/2) and phosphorylated CREB (P-CREB) in the hippocampus of the CUMS model groups decreased, but this decrease was reversed after treatment with 10 mg/kg of traxoprodil or 5 mg/kg of fluoxetine for 21 days and with 20 mg/kg or 40 mg/kg of traxoprodil for 7 days or 14 days. These results indicate that the inhibition of ERK1/2 and CREB phosphorylation induced by CUMS treatment can be alleviated by traxoprodil and fluoxetine. Therefore, the mechanism of traxoprodil may be related to the activation of the BDNF/ERK/CREB signaling pathway via upregulation of BDNF and phosphorylation of ERK1/2 and CREB.

On the other hand, FOXO transcription factors are regulators that mediate different physiological functions by regulating the expression of target genes [47]. FOXO subfamily genes include FOXO1, FOXO3, and other subtypes, which mainly regulate metabolism, antioxidant stress, and cell cycle progression, and are mostly related to cancer and diseases of the immune system [23, 48, 49]. The phosphatidylinositol-4,5-bisphosphate 3-kinase/protein kinase B (PI3K/Akt) signaling pathway plays an important role in cellular proliferation, cancer, and apoptosis PI3K activation phosphorylates and further activates Akt, promotes FOXO and Bim expression, and leads to cell apoptosis or division [50, 51]. Studies have demonstrated that the upregulation of Bim expression induced by the transcription factor FOXO1/3a in aging mice promotes mitochondrial disappearance and leads to cell apoptosis, indicating that the apoptotic factor Bim is involved in the apoptosis regulation of hippocampal neurons in the brain [52, 53]. Our study showed that although CUMS increased the expression of Akt, FOXO1, FOXO3a, and Bim in the hippocampus of mice, 20 mg/kg and 40 mg/kg of traxoprodil for 7 days or 14 days significantly reduced their expression. Thus, the antidepressant effect of traxoprodil in CUMS mice may be mediated by the Akt/FOXO/Bim signaling pathway.

A limitation of this study is that the protein expression of BDNF/ERK/CREB and Akt/FOXO/Bim signaling pathway in the hippocampus was measured only by western blot and immunohistochemistry. Furthermore, assessment of phosphorylated FOXO transcription factors and apoptosis-related experiments was not performed in this study. Additionally, further detailed studies are required that utilize inhibitors to block the expression of members of the BDNF/ERK/CREB and Akt/FOXO/Bim signaling pathways to confirm the mechanism by which traxoprodil exerts its antidepressant effects.

## 5. Conclusion

Overall, this study illustrated the antidepressant effects of traxoprodil at different concentrations after different durations of treatment. After 7 days and 14 days of treatment, the antidepressant effects of traxoprodil on the depressive mice increased with increasing dose, and both 20 and 40 mg/kg of traxoprodil produced rapid and strong antidepressant effects. However, after 21 days of administration, treatment with 10 and 20 mg/kg of traxoprodil exerted more pronounced antidepressant effects. The mechanism of antidepressant effects of traxoprodil may be closely related to the activation of BDNF/ERK/CREB and Akt/FOXO/Bim signaling pathway.

## Data Availability

Raw data is available from the corresponding author upon reasonable request.
